# p53 dynamics upon response element recognition explored by molecular simulations

**DOI:** 10.1038/srep17107

**Published:** 2015-11-24

**Authors:** Tsuyoshi Terakawa, Shoji Takada

**Affiliations:** 1 Department of Biophysics, Graduate School of Science, Kyoto University, Sakyo, Kyoto, Japan

## Abstract

p53 is a representative transcription factor that activates multiple target genes. To realize stimulus-dependent specificities, p53 has to recognize targets with structural variety, of which molecular mechanisms are largely unknown. Here, we conducted a series of long-time scale (totally more than 100-ms) coarse-grained molecular dynamics simulations, uncovering structure and dynamics of full-length p53 tetramer that recognizes its response element (RE). We obtained structures of a full-length p53 tetramer that binds to the RE, which is strikingly different from the structure of p53 at search. These structures are not only consistent with previous low-resolution or partial structural information, but also give access to previously unreachable detail, such as the preferential distribution of intrinsically disordered regions, the contacts between core domains, the DNA bending, and the connectivity of linker regions. We also explored how the RE variation affects the structure of the p53-RE complex. Further analysis of simulation trajectories revealed how p53 finds out the RE and how post-translational modifications affect the search mechanism.

p53 is a transcription factor that can activate^1^ or repress^2^ transcriptions to orchestrate multiple cellular processes (e.g. cell cycle arrest, DNA repair, apoptosis, and so on), depending on temporal status of a cell^3^,^4^. The genes that are induced in each process have different types of p53 recognition elements (REs) in its promoter region^5^. They have difference not only in their sequences but also in their structures: The REs are composed of two half-sites RRRCWWGYYY (R = A or G; W = A or T; Y = C or T) separated by a 0–21 bps long spacer^6^,^7^; the repressive REs prefer longer spacer, which implies biological relevance of the spacer length^4^,^8^. Despite intensive studies on p53 in the last three decades, molecular mechanisms of how p53 recognizes those varying REs are largely unknown.

The flexibility of p53 molecule makes the structural characterization of the p53-RE complex challenging. A large fraction of p53 in living cells forms a homo-tetramer^9^ in which each subunit is composed of five distinct regions [the N-terminal domain (NTD), the core domain (Core), the tetramerization (TET) domain, and the C-terminal domain (CTD)] together with one linker region between the Core and the TET domains^10^ (Fig. 1A). The NTD, the CTD, and the linker region that consist 40% of the molecule are intrinsically disordered regions^11^, which make the entire molecule extremely flexible.

Only partial or low-resolution structure has been resolved for the p53-RE complex. As for the partial structure, the crystal structures of the four Cores binding to the RE without spacer^12^ and with the 1-bp spacer^13^ were resolved. As for the low-resolution structure, Tidow *et al.* modeled a full-length p53 tetramer binding to the RE without spacer using cryo-electron microscopy^14^. So far, they have not resolved the structure of the full-length p53 tetramer binding to the RE with spacers, from which we can get insight into the molecular mechanism for the full-length p53 to recognize the varying REs.

Coarse-grained molecular dynamics simulation studies of our and other groups have greatly contributed to reveal molecular mechanisms of this flexible huge molecule. Khazanov *et al.* revealed inter-domain cross talks of p53 in the search mode at relatively low salt condition^15^ by utilizing native structure based one-bead-one-amino-acid protein model^16^, frozen three-beads-one-nucleotide DNA model, and simple protein-DNA interaction model that imposes only electrostatic interaction and excluded volume interaction. We extended this work^17^ in terms of the more sophisticated protein model^15^,^18^ and the flexible DNA model^19^, revealing that the conformation of p53 in the search mode at physiological salt condition is strikingly different from that in the recognition mode and that p53 slides along double strand (ds) DNA with its CTD^14^ (Fig. 1B (ii) (iv)).

In this work, as a natural extension of these previous works, we conducted coarse-grained molecular dynamics simulations in which structure based potentials are imposed to stabilize Core-RE complex structure^12^ in addition to the re-calibrated protein model^20^, the previously utilized DNA model^19^, and the simple protein-DNA interaction model. From these simulations, we obtained the structure of full-length p53 binding to the RE without and with 1-bp, 2-bp, and 10-bp spacers. These structures are not only consistent with previous low-resolution^14^ or partial structural information^12^,^21^, but also give access to previously unreachable detail, such as the preferential distribution of intrinsically disordered regions, the contacts between core domains, the DNA bending, and the connectivity of linker regions.

From analysis of the 100 trajectories in which initially free p53 finally binds to its RE (Fig. 1B), we found that, after one Core weakly (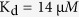) binds to the RE, the second and third Cores rapidly follow and inter-Core interaction cooperatively locks the complex structure (sequential recognition model). By this mechanism, the large conformational change from the search mode (Fig. 1B(ii)) to the recognition mode (Fig. 1B(iv)) is accomplished. Neutralizing negative charges in the CTD changes the search mechanism from 1D-sliding to 3D-diffusion, but still retaining essentially the same sequential recognition model and the final complex structure. This result indicates that post-translational modifications (e.g. acetylation^22^,^23^,^24^,^25^,^26^) of the CTD modulate the binding kinetics, necessitating the future *in vitro* and *in vivo* assays in which they monitor the relationship between post-translational modifications of the CTD and transactivation kinetics.

## Results and Discussions

### Coarse-grained molecular dynamics simulations of full-length p53

In the current work, we employed a coarse-grained representation, where, each amino acid in p53 is represented by one bead located at Cα position and each nucleotide is approximated by three beads, each representing phosphate, sugar, and base. We utilized the atomic interaction-based coarse-grained model version 2 (AICG2)^27^ for p53 and the 3SPN.1 model^19^ for DNA. For the intra-linker and inter-Core non-local interaction, we optimized non-local interaction parameters^20^. Previously, we showed that these optimizations are required to reproduce small angle X-ray scattering profile of p53 tetramer^20^. Following the previous works, we simply took electrostatic interaction and excluded volume interaction into account for p53-DNA interaction^15^,^17^. We put a positive charge on Lys, His, and Arg and a negative charge on Asp, Glu, and phosphate in dsDNA, and treated electrostatics using Debye-Hückel model. Parameters for p53-RE interaction were calibrated to reproduce an experimental dissociation constant (see “Parameter calibration for p53-RE interaction” in supporting information). All the simulations were performed with the coarse-grained molecular dynamics simulator, Cafemol^28^ (http://www.cafemol.org).

In the production simulations, we put full-length p53 and 100-bps long dsDNA in a sphere with a radius of 350 Å and with one end of the dsDNA being put at the center of the sphere. Two ends of the dsDNA were constrained. The RE was located 10-bps away from one end of the dsDNA. The p53 was initially put near but not on the other end of the dsDNA. We conducted 50 simulations by Langevin dynamics for 1-ms (see “Time mapping procedure” in supporting information; supplementary movie 1) with friction coefficient of 0.02 ps^−1^, temperature of 300 K, and salt concentration of 210 mM.

**Figure 1:**
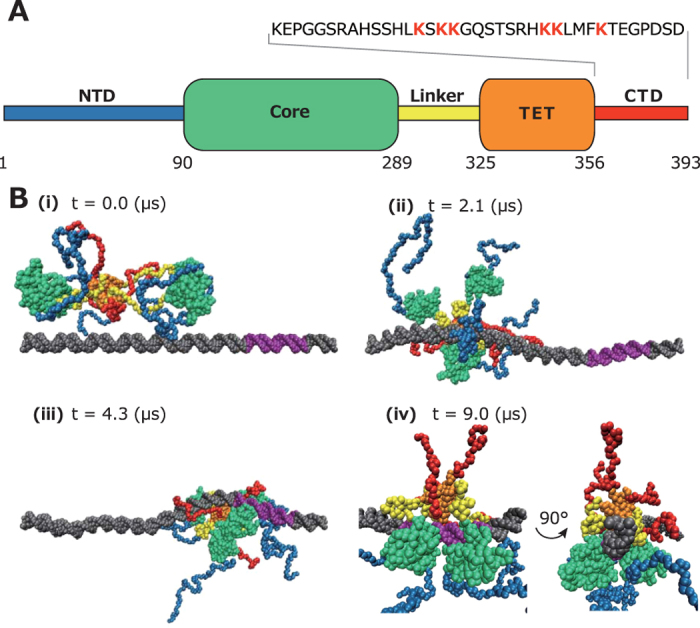
p53 domain structures and simulation snapshots. (**A**) The domain map of p53. The bars represent intrinsically disordered regions and the rounded rectangles represent folded domains. The numbers represent residue IDs. The amino acid sequence of the CTD is shown with acetylation sites colored red. (**B**) Representative snapshots taken from the coarse-grained molecular dynamics simulation trajectories. p53 slid along dsDNA (ii). After one of the Cores bound to the RE (iii), the other three Cores bound one by one, forming p53-RE complex (iv). dsDNA is colored grey and the RE is colored purple. The color-coding of p53 is the same as (**A**). In (iv), p53 in the recognition mode is viewed from two different orientations.

Right after the beginning of the simulation, p53 immediately bound to dsDNA near the initial position (Fig. 1B(i)). Then, p53 slid along dsDNA primarily using its CTDs (Fig. 1B(ii)). Two of the CTDs on average extended away from the DNA strand, which were indicated to capture the other DNA strand to accomplish a inter-segmental transfer (Terakawa *et al.* unpublished result). The NTDs stay away from the DNA strand, which makes the NTD easily accessible by other molecules to be recruited. In most of the simulations, p53 did not dissociate from dsDNA. After one of the Cores bound to the RE (Fig. 1B(iii)), the other three Cores sequentially bound one by one. The resulting p53-RE complex structure resembled previous cry-electron microscopy model (Fig. 1B(iv)): The entire molecule wraps around the DNA by its TETs, Linkers and Cores. This resemblance was not trivial because we only imposed structure-based interaction between the Core and the RE (TETs could stay the opposite side of the DNA strands, which did not occur in the simulations). This result shows that coarse-grained molecular dynamics simulations with models utilized here are able to reproduce the p53-RE complex structure from its free state.

### Simulations of p53 and the RE with spacers

**Figure 2:**
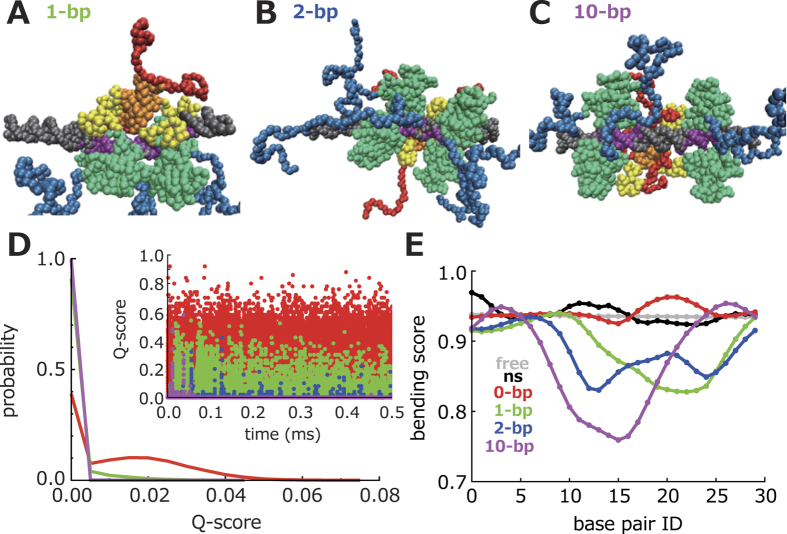
The p53-RE complex with varying spacer lengths. (**A–C**) Representative structures of the p53-RE complex in which the REs contain 1- (**A**), 2- (**B**), and 10- (**C**) bps spacers. (**D**) Probability distributions and time trajectories (inset) of Q-score of the inter-Core contacts of longitudinally aligned two Cores in which the RE contains 0- (red), 1- (green), 2- (blue), and 10-bps (magenta) spacers. (**E**) DNA bending scores vs base-pair IDs. The color assignment is the same as that of (**D**). We also plotted the scores of naked dsDNA (grey) and dsDNA to which p53 non-specifically binds (black).

Next, we address the p53-RE complex structure in which the RE has a spacer. For this purpose, we performed the simulations of the p53 and the RE with 1-, 2-, and 10-bps spacers in the 50-bps dsDNA. All the setups are the same as above except the dsDNA length, the RE structure, and the sphere radius (300 Å). In the several trajectories, the four core domains fully bound to the RE (Fig. 2A–C).

In the case of the 1-bp spacer, the longitudinally aligned two Cores extensively contact in the fully bound state (Fig. 2A). In the case of the 2-bp spacer, these two core domains hardly contact. Alternatively, the linker regions (yellow) are inserted between these two core domains (Fig. 2B). In the case of the 10-bp spacer, there is a larger space between these longitudinally aligned two Cores. Accordingly, the linker region (yellow) and the CTD (red) are located between these two Cores (Fig. 2C). It is interesting that not only the positions of core domains but also those of the linker region and the CTD are altered by the spacer length. From this result, we speculate that the spacer length affects the interactions of the linker region and the CTD with a different set of partner effector proteins of p53, and accordingly affects the downstream event, i.e. the transcriptional activation. It is interesting that the even same set of effectors can induce either transcriptional activation or repression on the different REs, suggesting different conformations of the p53-effector complexes on the varying RE^29^. Clarifying these effects is beyond the scope of this work, and should be approached in future.

In Fig. 2D, we plot probability distributions of Q-score between the longitudinally aligned two core domains, where the Q-score represents the ratio of the transiently formed contacts to the natively formed contacts^12^. From Fig. 2D, we can see again that the longitudinally aligned two Cores contact in the case of the 0-, and 1-bp spacer. Note that, in the crystal structure in which the four Cores bind to the RE with a 1-bp spacer, these two Cores contact by deforming dsDNA, consistent with this simulation result. From inset of Fig. 2D (time course of Q-score), we see that these two Cores intermittently contact in the case of the 2-bps spacer. In the case of the 10-bps spacer, these two Cores do not contact at all. The difference of interaction between core domains depending on the spacer length is supposed to compromise the cooperativity and the affinity, which might contribute to the RE selectivity^30^. Previously, the similar speculation was inferred from the result of the more detailed atomic simulation^31^,^32^ using a partial structure of p53.

We also noticed that binding of p53 to the RE deforms dsDNA in the simulations. To quantify the degree of the deformation, we defined a score 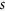 that takes unity for straight dsDNA and decreases as dsDNA bends (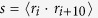
where 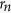 is the vector from 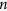-th to 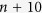-th sugar particle and 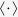 represents an ensemble average). We plotted 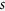 against base-pair IDs in Fig. 2E. Note that we fixed two ends of dsDNA, which must have reduced the degree of bending and thus the absolute bent angle is not of our interest. Instead, we focus on the relative bending. Re-calibration of the model parameters may be required to quantitatively estimate the absolute bent angle and must be addressed in future. By comparing the grey and black lines in Fig. 2E, we see that dsDNA slightly bends when p53 non-specifically binds to dsDNA. In the case of the 0-bp spacer, 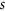 is higher around the RE than the naked dsDNA, suggesting that the p53 binding makes the dsDNA straighter. This result is consistent with the crystal structure in which we observe no discernible bend of the dsDNA^12^. In the case of the 1-bp spacer, 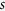 is lower around the RE than naked dsDNA, suggesting that p53 binding makes the dsDNA bent to accommodate the spacer^13^. In the case of the 2-bps spacer, 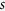 shows two basins, suggesting that the two pairs of the transversely aligned Cores independently make dsDNA bent. In the case of the 10-bps spacer, 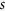 is lowest. Taken together, these results suggest that the extent to which the dsDNA bends highly depends on the spacer length. Previously, Nussinov *et al.* addressed the questions of the Core binding-induced DNA bending and the inter-Core contacts with the full-atomic model^32^,^33^. In this work, we extended these works to the full-length tetrameric p53 by taking advantage of the coarse-graining.

### Conformations of p53 on the response element

**Figure 3:**
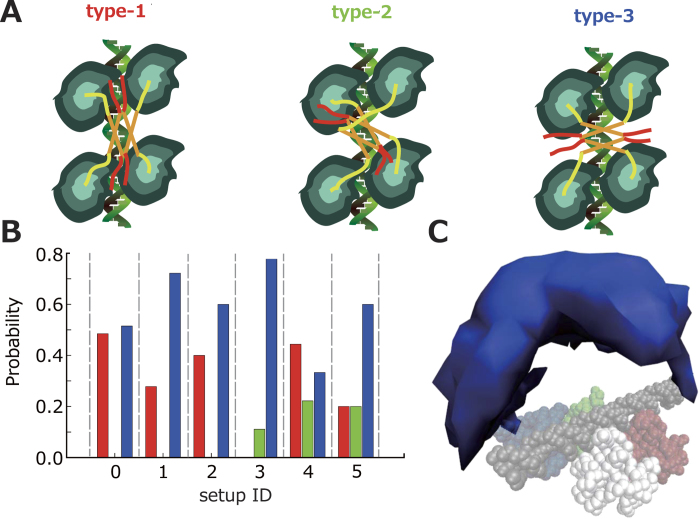
Conformations of full-length p53 bound on the RE. (**A**) A definition of the three types in which the four Cores are tethered to the TET. The color-coding of p53 is the same as Fig. 1A. The DNA is colored light green. (**B**) Probabilities of the three types (type-1 in red, type-2 in green, and type-3 in blue) from the six sets of the simulations with different setups (Table 1). (**C**) An iso-surface (0.001) of a spatial probability distribution of the center of the Core (blue) that forms dimers with the fixed one (white). The Cores that bind to the RE are depicted by four different colors (white, red, blue, and green). The dsDNA is colored grey.

In full-length p53 tetramer, the TET forms a dimer of dimers^14^. In addition, the four Cores bind to the RE also in the form of a dimer of dimers. The Core and the TET are tethered by a flexible linker region, resulting in the three combinatorial types, as shown in Fig. 3A. To reveal the most dominant type, we calculated probabilities of these types using a final snapshot of the simulations listed in Table 1 (Fig. 3B). This figure shows that the type-3 is the most dominant type in all the setups except the setup-5. By comparing the setup-2 and the setup-3, we can also see that the dominant type does not depend on a dsDNA length. Interestingly, the type-2 is not observed when there is no spacer.

Next, we tried to reveal the reason why the type-3 is the most dominant and why the type-2 is unlikely when there is no spacer. For this purpose, we performed the additional simulations in which one of the Cores is fixed on the RE and interactions between the other Cores and the RE are turned off. From these simulations, we calculated the spatial probability distribution of the core domain that forms dimers with the fixed core domain. In Fig. 3C, we show a relatively high probability (0.001) iso-surface. From this figure, we can see that the high probability iso-surface is around the binding site next to the fixed Core, indicating that this topological constraint is one of the reasons why the type-3 is the most dominant and why the type-2 is unlikely.

### Analysis of the search process

**Figure 4:**
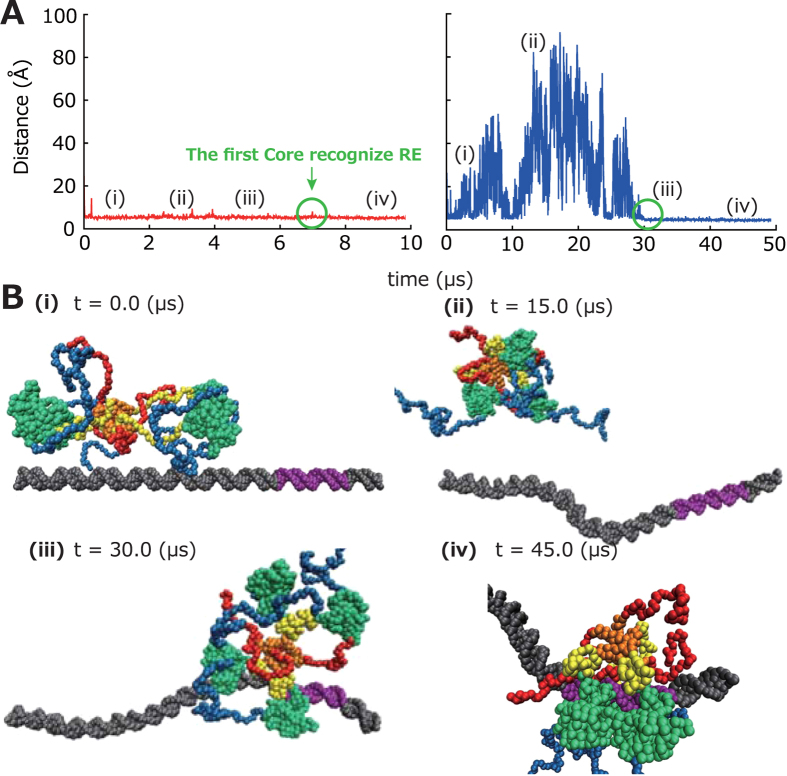
Effects of charge neutralization in CTD. (**A**) The time trajectories of nearest distance between p53 and dsDNA in the simulations in which the charges of CTD is not neutralized (p53_0_; left) and is neutralized (p53_n_; right). The black circles indicate the time when the first core recognized the RE. The roman numerals represent the snapshot IDs in Fig. 1B (left) and Fig. 4B (right). (**B**) Representative snapshots taken from the trajectories for p53_n_. p53 diffused around dsDNA (ii). After one of the Cores bound to the RE (iii), the other three Cores bound one by one, forming p53-RE complex (iv).

As we showed above, the coarse-grained molecular dynamics simulation utilized here is able to reproduce the fully bound state from the free state. Therefore, we next focus on the search process. Fig. 4A (left) depicts a representative time course of the nearest distance between p53 and dsDNA. This figure indicates that p53 is always in close contact with dsDNA, which implies sliding along dsDNA. This result is consistent with the previous work in which slightly different model of p53 was utilized^17^, supporting the robustness of this result.

The previous study^17^ suggested that p53 slides along dsDNA with its positively charged CTD. To confirm the importance of the positively charged CTD for the sliding, we neutralized the charges of the six Lys residues in the CTD and repeated the 50 simulations (supplementary movie 2). Note that this situation is realized *in vivo* by post-translational modifications, namely acetylation which neutralized six Lys residues in the CTD^22^,^23^,^24^,^25^,^26^. Fig. 4A (right) and Fig. 4B show that neutralized p53 is far from dsDNA and diffuses three dimensionally at some earlier part of the trajectory, strongly corroborating that the positive charges in the CTD are critical for p53 sliding^17^,^34^.

In the structural point of view, the initial structure (Fig. 4B (i)) is the same as the case of un-neutralized p53 (p53_0_ hereafter). After beginning of the simulation, the neutralized p53 (p53_n_ here after) did not immediately bind to the dsDNA. In most of the trajectories, the p53_n_ freely diffused three dimensionally (Fig. 4B (ii)). Thus, the acetylation (or any post-translational modifications) of the CTD might alter the dominant search mechanism from “sliding” to “3D diffusion” (see “dominant search mechanism” in supporting information). Previously, McKinney *et al.* proposed that the modification of the CTD switches the search mechanism^26^, which is consistent with our result. After one of the core domains bound to the RE (Fig. 4B (iii)), the other three core domains sequentially bound one by one, forming the complete p53-RE complex, which seemingly is not different from that of p53_0_ (Fig. 4B (iv)). Thus, the recognition mechanism after the search is essentially the same as that of p53_0_.

In these simulations, the association rate constant of p53_n_ to RE is 2.8 higher than that of p53_0_. The effect of charge neutralization on the search speed should be dependent on the length of the dsDNA flanking RE. Our preliminary analysis indicates that the association rate constant of p53_0_ might be higher than that of p53_n_ also *in vivo* (see “Association rate constant analysis” in supporting information).

### A binding kinetics of core domains

**Figure 5:**
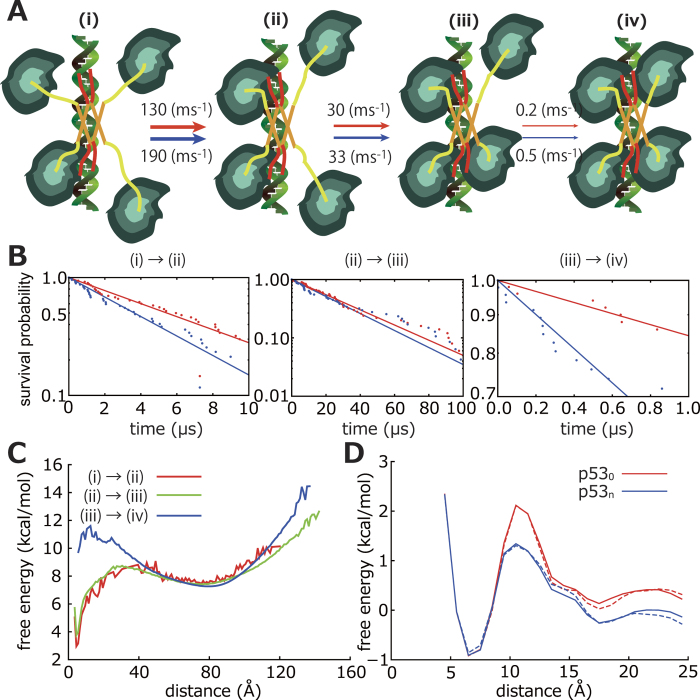
The analysis of the sequential binding of the Core to the RE. (**A**) The scheme of the four Cores binding to the RE with rate constants (p53_0_ in red and p53_n_ in blue). (**B**) The fractions of the 1- (left), 2- (center), and 3- (right) Core bound states against duration. (**C**) The approximate free energy along the distance between the RE and the unbound Core that is the nearest from the RE in the 1- (red), 2- (blue), and 3- (green) Core bound states. (**D**) Free energy profiles along the reaction coordinate. The solid and dashed lines represent free energy profiles from the two independent simulations with the same setup. The similarity of these lines shows the convergence of the simulation.

In the simulations of p53 binding to the RE (Fig. 4), we saw that, after one of the Cores bound to the RE, the other Cores sequentially bound one by one (Fig. 5A). Here, we analyse kinetics of individual processes for p53_0_ and p53_n_. Fig. 5B shows the fractional survival of each intermediate as a function of time, where the y-axis is in a logarithmic scale. All the kinetics can be fit with single exponential functions, 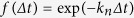 where 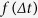 and 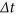 are the population and the duration, respectively. 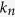 is the rate constant of the transition from the 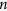
- to 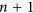-th Core bound state. Both for p53_0_ and p53_n_, the orders of magnitude of the 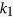, 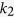 and 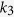 are 100, 10 and 0.1 ms^−1^, respectively. Note again that the time-scale was mapped using the diffusion of the core domain. This result indicates that the latter transitions are slower and thus the rate-limiting step is the transition from the 3- to the 4-Core bound state (Fig. 5A). We also find that, although 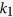 and 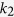 are not significantly altered by acetylation of the CTD, 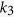 of p53_n_ is about 3 times larger than that of p53_0_ (Fig. 5A). Yet, we should note that, in a genome-scale search, a time-scale of recognition (binding of the second, third and forth core domains to the RE) is much shorter than that of a search (binding of the first core domain to the RE), not supporting the strong inhibitory effect of the CTD on the Core binding.

To reveal the reason why 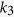 is smaller than 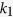 and 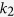, for p53_0_, we plotted approximate free energy curves (more precisely, the potentials of mean force) along nearest distance between an unbound core domain and the RE in a 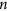-core bound state (Fig. 5C). Note that these approximate free energy curves were calculated from non-equilibrium simulations described above. Because most of the trajectories did not reach the 4-Core bound state due to the limited sampling, there is no deep minimum at the short distance in the plot (blue line in Fig. 5C). Fig. 5C shows that the unbound Core tends to stay 80 Å away from the RE in the 3-Core bound state (blue). At this long distance, there is no significant direct interaction between the Core and the RE. The detailed analysis indicates that the binding of three Cores provides topological constraint for the accessible range of the 4-th core domain, making the binding of the 4-th Core hard (see “Accessible range of the 4-th core domain” in supporting information). It is interesting to note that p53 can induce transcriptions of genes even if there are binding sites only for three Cores^8^.

Due to the limited sampling of the 4-Core bound state, the approximate free energy in Fig. 5C does not provide information on the free energy barrier height between the 3-and 4-core bound states. This barrier is caused by relatively short-range interaction of the unbound core domain with the other bound core domains (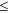
25 Å where the electrostatic energy between two unit charges drops to 10% of that at 1 Å in 210 mM ion). To investigate the free energy barrier height, we performed replica exchange umbrella sampling (REUS) simulations^35^. In Fig. 5D, we show the free energy curves for p53_0_ (red) and p53_n_ (blue). The free energy barrier height between the 3- and 4-Core bound states of p53_n_ is 1.5 kcal/mol lower than that of p53_0_. We also see that the bound state is more stable than the unbound state by about 1.0 kcal/mol. This low stability of the bound state is due to fixed dsDNA since the bound state is stable for 1-ms in the 10 independent simulations with the flexible dsDNA model (data not shown; supplementary movie 3).

Currently, there is no experimental report on this binding kinetics and thus the current results should be viewed as predictions. The predictions can be tested experimentally for example by single molecule FRET measurements. In addition to the effect of acetylation on the binding kinetics described above, p53 bearing acetylated CTDs recruits other proteins such as a histon-acetyl-transferase^36^. These multiple effects of acetylation might make interpretations of *in vitro* and *in vivo* experiments difficult^37^.

## Conclusion

In this work, we performed long time-scale coarse-grained molecular dynamics simulations in which unbound p53 searches and recognizes its target RE. These simulations revealed the quaternary structures of p53 on the various REs. These structures are not only consistent with previous low-resolution or partial structural information, but also give access to previously unreachable detail, such as dynamics of the core domains on the RE, preferential positioning of intrinsically disordered domains, and connectivity of linker regions. The result also indicates that post-translational modifications of the CTD modulate the binding kinetics, necessitating the future *in vitro* and *in vivo* assays in which they monitor the relationship between post-translational modifications of the CTD and transactivation kinetics.

The current work left following challenges as well. Previously, it is reported that the modification in the CTD alters the conformation of the p53 entire molecule, and thereby regulates its RE binding activity, which is not reproduced in the current model. In addition, the model utilized here cannot be applied for the cancer mutations that destabilize the Core structure because the current model is based on the native stable structure. The modeling of the destabilized structures would be the next step to make the model relevant to medical research. It would also be interesting to reveal the search mechanism of p53 attached to the main effector proteins, such MDM2, p300/CBP and so on. These should be approached in near future.

## Methods

### A protein coarse-grained model for folded domains

For a coarse-grained model of folded domains, we utilized the AICG2 model developed by Li *et al.*^27^. Each coarse-grained particle located on a 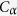 atom represents an amino acid (refer to original paper^27^ for detail). The native structure based interaction stabilizes the folded structures. The AICG2 model was utilized for the Core (residues 91–289) and the TET domain (residues 326–356).

### A protein model for the NTD and the CTD

For a coarse grained model of the intrinsically disordered domains [i.e. NTD (residues 1–90) and CTD (residues 357–393)], we utilized statistical potentials for virtual bond angles and dihedral angles. These statistical potentials were constructed from loop structures in the PDB in a sequence dependent way. In our previous work, this approach reasonably reproduced the SAXS profiles and NMR residual dipolar couplings of the p53 NTD^18^. Refer to the original paper for detail^18^. The ignorance of sequence specific non-local contact interaction makes it hard to represent binding induced conformational changes. Specific to NTD and CTD of p53, which are highly hydrophilic, we do not consider non-local contact play crucial roles in the current systems.

### A protein model for linker region

The potential energy function for the linker region is


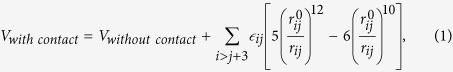


where 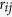 is the distance between 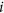
- and 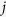-th particles. 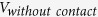 is the same potential energy function as that of NTDs and CTDs described above. In our previous work, we determined 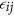
s and 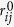
s so that a contact probability map of the linker region obtained using an all atom molecular dynamics simulation was reproduce by the coarse-grained molecular dynamics simulation. Refer to the original paper for detail^20^.

### A DNA model

For a DNA model we used the 3SPN.1 model developed by the de Pablo’s group^19^. This model was calibrated to reproduce ion concentration dependency of melting temperature, a persistence length, and heat capacity of dsDNA. Each nucleotide is represented by three coarse-grained particles (sugar, phosphate, and base). Refer to the original paper for detail^19^.

### Inter-chain interaction model

We imposed excluded volume and electrostatic interactions between different subunits of p53 and between p53 and dsDNA. The electrostatic interaction was modeled by the Debye-Hückel theory. Refer to the previous work for detail^17^. In addition, we imposed native structure^12^ based interaction between the Core and the RE as described above. We also imposed native structure based interaction between the Cores. The interaction strength is determined to reproduce the experimental dissociation constant. Refer to the original paper for detail^20^.

### Preparation of the p53 initial structure

We prepared the initial structure of the p53 using Modeller^38^. We used 2XWR^39^ and 1AIE^21^ as template structures for the core (residues 91–289) and the TET (residues 326–356) domains, respectively, and modeled the NTD (residues 1–90), the CTD (residues 357–393), and the linker region (residues 290–325) as random coils.

### Replica exchange umbrella sampling simulations

To calculate the free energy profile of the process in which the Core binds to the RE, we conducted replica exchange umbrella sampling simulations. In this method, pairs of replicas of systems with different biasing potentials were exchanged after every 10-ns. For the biasing potentials, the distance between the Core and the RE was chosen as the reaction coordinate, 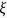. Then, the harmonic biasing potential,


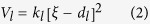


was imposed to 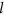-th replica. 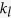 was set to 1.0 kcal/mol·Å and 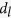 was set to the value from 4.0 to 27.0 Å at intervals of 1 Å. In these simulations, The 50 bps long dsDNA and three Cores on it were fixed. We conducted these simulations by Langevin dynamics for 1-ms with friction coefficient of 0.02 ps^−1^, temperature of 300 K, and ion concentration of 210 mM.

The potential of mean force as a function of the reaction coordinate 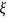 is given by


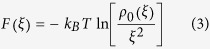


where 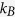 is the Boltzmann constant, 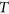 is temperature, and 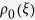 is unbiased probability distribution of 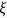 obtained by the weighted histogram analysis method^40^.

## Additional Information

**How to cite this article**: Terakawa, T. and Takada, S. p53 dynamics upon response element recognition explored by molecular simulations. *Sci. Rep.* **5**, 17107; doi: 10.1038/srep17107 (2015).

## Supplementary Material

Supplementary Information

Supplementary Movie 1

Supplementary Movie 2

Supplementary Movie 3

## Figures and Tables

**Table 1:** Coarse-grained simulation setups.

ID	CTD	DNA length (bps)	Spacer (bps)
1	un-neutralized	100	0
2	neutralized	100	0
3	neutralized	50	0
4	neutralized	50	1
5	neutralized	50	2
6	neutralized	50	10

## References

[b1] Funk W. D., Pak D. T., Karas R. H., Wright W. E. & Shay J. W. A transcriptionally active DNA-binding site for human p53 protein complexes. Mol. Cell. Biol. 12, 2866–2871 (1992). 1588974 10.1128/mcb.12.6.2866-2871.1992 PMC364481

[b2] Ginsberg D., Mechta F., Yaniv M. & Oren M. Wild-type p53 can down-modulate the activity of various promoters. Proc. Natl. Acad. Sci. USA 88, 9979–9983 (1991). 1946467 10.1073/pnas.88.22.9979 PMC52850

[b3] Levine A. J. & Oren M. The first 30 years of p53: growing ever more complex. Nat. Rev. Cancer 9, 749–758 (2009). 19776744 10.1038/nrc2723 PMC2771725

[b4] Riley T., Sontag E., Chen P. & Levine A. Transcriptional control of human p53-regulated genes. Nat. Rev. Mol. Cell Biol. 9, 402–412 (2008). 18431400 10.1038/nrm2395

[b5] Weinberg R. L., Veprintsev D. B., Bycroft M. & Fersht A. R. Comparative Binding of p53 to its Promoter and DNA Recognition Elements. J. Mol. Biol. 348, 589–596 (2005). 15826656 10.1016/j.jmb.2005.03.014

[b6] el-Deiry W. S., Kern S. E., Pietenpol J. A., Kinzler K. W. & Vogelstein B. Definition of a consensus binding site for p53. Nat. Genet. 1, 45–49 (1992). 1301998 10.1038/ng0492-45

[b7] Wang B., Xiao Z. & Ren E. C. Redefining the p53 response element. Proc. Natl. Acad. Sci. USA 106, 14373–14378 (2009). 19597154 10.1073/pnas.0903284106 PMC2709670

[b8] Jordan J. J. *et al.* Noncanonical DNA Motifs as Transactivation Targets by Wild Type and Mutant p53. PLoS Genetics 4, e1000104 (2008). 18714371 10.1371/journal.pgen.1000104 PMC2518093

[b9] Gaglia G., Guan Y., Shah J. V. & Lahav G. Activation and control of p53 tetramerization in individual living cells. Proc. Natl. Acad. Sci. USA 110, 15497–15501 (2013). 24006363 10.1073/pnas.1311126110 PMC3780836

[b10] Joerger A. C. & Fersht A. R. Structural Biology of the Tumor Suppressor p53. Annu. Rev. Biochem. 77, 557–582 (2008). 18410249 10.1146/annurev.biochem.77.060806.091238

[b11] Bell S., Klein C., Müller L., Hansen S. & Buchner J. p53 Contains Large Unstructured Regions in its Native State. J. Mol. Biol. 322, 917–927 (2002). 12367518 10.1016/s0022-2836(02)00848-3

[b12] Chen Y., Dey R. & Chen L. Crystal Structure of the p53 Core Domain Bound to a Full Consensus Site as a Self-Assembled Tetramer. Structure 18, 246–256 (2010). 20159469 10.1016/j.str.2009.11.011 PMC2824536

[b13] Chen Y. *et al.* Structure of p53 binding to the BAX response element reveals DNA unwinding and compression to accommodate base-pair insertion. Nucleic Acids Res. 41, 8368–8376 (2013). 23836939 10.1093/nar/gkt584 PMC3783167

[b14] Tidow H. *et al.* Quaternary structures of tumor suppressor p53 and a specific p53 DNA complex. Proc. Natl. Acad. Sci. USA 104, 12324–12329 (2007). 17620598 10.1073/pnas.0705069104 PMC1941468

[b15] Khazanov N. & Levy Y. Sliding of p53 along DNA Can Be Modulated by Its Oligomeric State and by Cross-Talks between Its Constituent Domains. J. Mol. Biol. 408, 335–355 (2011). 21338609 10.1016/j.jmb.2011.01.059

[b16] Clementi C., Nymeyer H. & Onuchic J. N. Topological and energetic factors: what determines the structural details of the transition state ensemble and ‘en-route’ intermediates for protein folding? An investigation for small globular proteins. J. Mol. Biol. 298, 937–953 (2000). 10801360 10.1006/jmbi.2000.3693

[b17] Terakawa T., Kenzaki H. & Takada S. p53 searches on DNA by rotation-uncoupled sliding at C-terminal tails and restricted hopping of core domains. J. Am. Chem. Soc. 134, 14555–14562 (2012). 22880817 10.1021/ja305369u

[b18] Terakawa T. & Takada S. Multiscale Ensemble Modeling of Intrinsically Disordered Proteins: p53 N-Terminal Domain. Biophys J. 101, 1450–1458 (2011). 21943426 10.1016/j.bpj.2011.08.003 PMC3177054

[b19] Sambriski E. J., Schwartz D. C. & de Pablo J. J. A mesoscale model of DNA and its renaturation. Biophys. J. 96, 1675–1690 (2009). 19254530 10.1016/j.bpj.2008.09.061 PMC2717267

[b20] Terakawa T., Higo J. & Takada S. Multi-scale Ensemble Modeling of Modular Proteins with Intrinsically Disordered Linker Regions: Application to p53. Biophys J. 107, 721–729 (2014). 25099811 10.1016/j.bpj.2014.06.026 PMC4129485

[b21] Mittl P. R., Chene P., Grutter M. G., Gu W. & Roeder R. G. Crystallization and structure solution of p53 (residues 326–356) by molecular replacement using an NMR model as template. Acta Crystallogr. D Biol. Crystallogr. 54, 86–89 (1998). 9761820 10.1107/s0907444997006550

[b22] Activation of p53 sequence-specific DNA binding by acetylation of the p53 C-terminal domain. **90**, 595–606 (1997). 10.1016/s0092-8674(00)80521-8 9288740

[b23] Lill N. L., Grossman S. R., Ginsberg D., DeCaprio J. & Livingston D. M. Binding and modulation of p53 by p300/CBP coactivators. Nature 387, 823–827 (1997). 9194565 10.1038/42981

[b24] Tang Y., Zhao W., Chen Y., Zhao Y. & Gu W. Acetylation is indispensable for p53 activation. Cell 133, 612–626 (2008). 18485870 10.1016/j.cell.2008.03.025 PMC2914560

[b25] Gu B. & Zhu W.-G. Surf the post-translational modification network of p53 regulation. Int. J. Biol. Sci. 8, 672–684 (2012). 22606048 10.7150/ijbs.4283 PMC3354625

[b26] McKinney K., Mattia M., Gottifredi V. & Prives C. p53 linear diffusion along DNA requires its C terminus. Mol. Cell 16, 413–424 (2004). 15525514 10.1016/j.molcel.2004.09.032

[b27] Li W., Terakawa T., Wang W. & Takada S. Energy landscape and multiroute folding of topologically complex proteins adenylate kinase and 2ouf-knot. Proc. Natl. Acad. Sci. USA 109, 17789–17794 (2012). 22753508 10.1073/pnas.1201807109 PMC3497823

[b28] Kenzaki H. *et al.* CafeMol: A Coarse-Grained Biomolecular Simulator for Simulating Proteins at Work. J. Chem. Theory Comput. 7, 1979–1989 (2011). 10.1021/ct2001045 26596457

[b29] Kasper L. H., Thomas M. C., Zambetti G. P. & Brindle P. K. Double null cells reveal that CBP and p300 are dispensable for p53 targets p21and Mdm2but variably required for target genes of other signaling pathways. Cell Cycle 10, 212–221 (2014). 21220944 10.4161/cc.10.2.14542 PMC3048793

[b30] Pan Y., Tsai C.-J., Ma B. & Nussinov R. Mechanisms of transcription factor selectivity. Trends Genet. 26, 75–83 (2010). 20074831 10.1016/j.tig.2009.12.003 PMC7316385

[b31] Pan Y. & Nussinov R. Structural basis for p53 binding-induced DNA bending. J. Biol. Chem. 282, 691–699 (2007). 17085447 10.1074/jbc.M605908200

[b32] Pan Y. & Nussinov R. p53-Induced DNA bending: the interplay between p53-DNA and p53-p53 interactions. J. Phys. Chem. B 112, 6716–6724 (2008). 18461991 10.1021/jp800680w PMC2755056

[b33] Pan Y. & Nussinov R. Cooperativity dominates the genomic organization of p53-response elements: a mechanistic view. PLoS Comput. Biol. 5, e1000448 (2009). 19629163 10.1371/journal.pcbi.1000448 PMC2705680

[b34] Tafvizi A., Huang F., Fersht A. R., Mirny L. A. & van Oijen A. M. A single-molecule characterization of p53 search on DNA. Proc. Natl. Acad. Sci. USA 108, 563–568 (2011). 21178072 10.1073/pnas.1016020107 PMC3021058

[b35] Sugita Y., Kitao A. & Okamoto Y. Multidimensional replica-exchange method for free-energy calculations. J. Chem. Phys. 113, 6042 (2000).

[b36] Barlev N. A. *et al.* Acetylation of p53 activates transcription through recruitment of coactivators/histone acetyltransferases. Mol. Cell 8, 1243–1254 (2001). 11779500 10.1016/s1097-2765(01)00414-2

[b37] Espinosa J. M. & Emerson B. M. Transcriptional regulation by p53 through intrinsic DNA/chromatin binding and site-directed cofactor recruitment. Mol. Cell 8, 57–69 (2001). 11511360 10.1016/s1097-2765(01)00283-0

[b38] Martí-Renom M. A. *et al.* Comparative protein structure modeling of genes and genomes. Annu. Rev. Biophys. Biomol. Struct. 29, 291–325 (2000). 10940251 10.1146/annurev.biophys.29.1.291

[b39] Natan E. *et al.* Interaction of the p53 DNA-Binding Domain with Its N-Terminal Extension Modulates the Stability of the p53 Tetramer. J. Mol. Biol. 409, 358–368 (2011). 21457718 10.1016/j.jmb.2011.03.047 PMC3176915

[b40] Rosenbergl J. M. The weighted histogram analysis method for free-energy calculations on biomolecules. I. The method. J. Comput. Chem. 8, 1011–1021 (1992).

