# Supplementary material for: p53 dynamics upon response element recognition explored by molecular simulations
Source: Sci Rep. 2015 Nov 24;5:17107. doi: 10.1038/srep17107 (PMC4656996; doi:10.1038/srep17107)
Supplement: Supplementary Information [file srep17107-s1.pdf]

Supporting information for p53 dynamics upon response element recognition explored by molecular simulations

Authors: Tsuyoshi Terakawa[1] and Shoji Takada[1*]

Affiliations:
[1]Depart of Biophysics, Kyoto University, Sakyo, Kyoto, Japan
*To whom correspondence should be addressed: takada@biophys.kyoto-u.ac.jp

SI text:

Parameter calibration for p53-RE interaction

The energy function for an interaction between a core domain (Core) and a response element (RE) is defined as

$$V_{specific} = V_{non-specific} + \sum_{i>j+3}^{native\ contact} \epsilon \left[5\left(\frac{r_{ij}^0}{r_{ij}}\right)^{12} - 6\left(\frac{r_{ij}^0}{r_{ij}}\right)^{10} \right], \quad \text{(S1)}$$

where the summation is taken for protein coarse-grained particles i and DNA coarse-grained particles j that makes a contact in the crystal structure[1] (PDB ID: 3KMD). We consider that these two coarse-grained particles make a contact when one of the heavy atoms represented by the i-th coarse-grained particle is within 6.5 Å from those represented by the j-th coarse-grained particle. The r_{ij}^0 is the distance between the two coarse-grained particles in the crystal structure.

The coefficient ϵ modulates affinity between p53 and the RE. We tuned values of ϵ based on the experimental data; the dissociation constant of the single wild-type core domain of p53 from the RE was measured as 14 μM[2]. To tune the ϵ, we performed a series of coarse-grained molecular dynamics simulations using Langevin dynamics for 0.1 ms with a friction coefficient of 0.02 ps^{-1}. The simulation system put one p53 core domain and 20-bp long double strand (ds) DNA that includes the RE into a spherical wall (Fig. S1A). The length of dsDNA, the ion concentration, and the temperature were set to the same values as those in the experiment[2]. The coarse-grained molecular dynamics simulations were repeated for various ϵ values, ranging from 0.20 to 0.29 [kcal/mol] with a step of 0.01.

Fig. S1B shows representative time courses of the nearest distance between the p53 core domain and the

RE for three ϵ values. In this range of ϵ, the core domain repeatedly associates to and dissociates from the RE. The number of observed dissociation events decrease as the ϵ increases. The probability distribution of interaction energy (Fig. S1C) takes bimodal shape with a broad peak around -2.5 kcal/mol (bound state) and a sharp peak around zero energy (unbound state).

From each trajectory, we calculated dissociation constants according to the equation,

$$K_d = \frac{C(1-f_b)^2}{f_b}, \tag{S2}$$

where C is a concentration of the core domain (0.4 mM) and f_b is a fraction of a bound state. We determined $\epsilon = 0.26$ so that the calculated dissociation constant of the core domain from the RE was essentially same as that of the experiment[2] (Fig. S1D).

Time mapping procedure

To estimate the length of each simulation, first, we calculated the diffusion constant of the core domain of p53 in the coarse-grained molecular dynamics simulation. Then, we compared this value with the theoretical diffusion constant of a sphere with a radius of 25.0 Å (about the size of the core domain) from the Stokes-Einstein equation. From this comparison, we considered that the single step in coarse-grained molecular dynamics simulation corresponds to 1-ps. We used the diffusion constant of the core domain of p53 for time mapping, because time-scale is most important in this study when the kinetics of the core domain motion is investigated.

Dominant search mechanism

To reveal the dominant search mechanism in the 50 trajectories, we plotted probability distributions of the nearest distance between p53 and dsDNA (Fig. S2). We see that $p53_n$ exhibits a broad distribution over 10-250 Å, which clearly indicates the "3D diffusion" mechanism. On the contrary, $p53_0$ does not show any significant

distribution in the same range, indicating the "sliding" mechanism. The significant difference between $p53_0$ and $p53_n$ shows that the simulation time is enough to statistically distinguish these two diffusion modes. Thus, the charge neutralization of 6 lysine residues in the p53 CTD alters the dominant search mechanism from "sliding" to "3D diffusion".

Association rate constant analysis

Here, we discuss its impact on search kinetics on the basis of the Berg's classic theory[3]. We begin by supposing that a transcription factor finds its RE via purely 3D diffusion. We assign this diffusion-limited association rate constant as k_a. Now, we incorporate two additional factors. First, we suppose that the RE is flanked by some non-specific dsDNA and the transcription factor can slide along the non-specific dsDNA for an average length of $\langle L \rangle$ bps. Thus, the binding to any positions of dsDNA within $\langle L \rangle$ bps around the RE leads to association to the RE. This so-called antenna effect speeds up association. Second, we suppose that, in a biochemical bulk assay, a non-specific dsDNA with the concentration $[DNA]$ is usually added as a competitor[4]. Once a transcription factor binds to the competitor, it is trapped for a while, slowing down association to the RE. In this circumstance, the association rate constant, k_{on}, to the RE in the presence of an excess dsDNA can be written as,

$$k_{on} = \frac{k_a}{1 + K_a[DNA]} \langle L \rangle, \tag{S3}$$

where K_a is an association equilibrium constant to the competitor. We can rewrite this equation as,

$$k_{on} = k_a \frac{\langle L \rangle}{1 + \frac{\tau_a}{\tau_d}}, \tag{S4}$$

where τ_a (τ_d) is life time of the dsDNA-bound (unbound) state. This equation can be used to interpret the association rate constant to the RE in genomic DNA. Large portion of the genomic DNA that is distant from the RE more than $\langle L \rangle$ bps is apparently competitor dsDNA. In this equation, we can consider $\langle L \rangle$ as a promoting factor and $\left(1 + \frac{\tau_a}{\tau_d}\right)$ as an inhibitory factor for the association rate constant to the RE.

Previously, life times τ_a and τ_d were measured *in vivo*[5]. Using these values, we can estimate the inhibiting factor $\left(1+\frac{\tau_a}{\tau_d}\right)$ *in vivo*. In the experiment, they measured the life times of the full-length p53 and that lacking the CTD. As a rough estimate, it is reasonable that the construct lacking the CTD corresponds to the $p53_n$ in the simulation because this construct lacks positive charges in the CTD. For the full-length p53 and that lacking the CTD, the inhibiting factors are 1.2 and 1.1, respectively. Thus, in terms of the inhibiting factors, these two constructs are similar. On the contrary, we can assume that an average sliding length of full-length p53 may be longer, because τ_a of full-length p53 ($\tau_a = 1.72\ [s]$) is longer than that lacking the CTD ($\tau_a = 0.2\ [s]$). Considering these factors together, the association rate constant of $p53_0$ should be higher than that of $p53_n$ *in vivo*.

Accessible range of the 4-th core domain

To get insight into the reason why it is difficult for the 4-th Core to bind to the RE, we plot a two-dimensional probability distribution of two angles, α and β, in Fig. S3. The angles α and β are defined by the relative positions of unbound core domain, the TET domain, and the bound core domain. The red circle in Fig. S3 indicates the 4-Core bound state. The Fig. S3A suggests that the 0-Core bound state samples broad range on this plane and that the 1-Core bound state is easily accessible. On the contrary, Fig. S3B shows that limited angles are sampled in the 3-Core bound state. Importantly, the angles for the 4-Core bound state are hardly sampled. Thus, binding of the other three Cores provides strong restriction to the accessible range of the 4-th Core, and thus the binding of the 4-th Core is harder.

Reference:

1. Chen, Y., Dey, R. & Chen, L. Crystal Structure of the p53 Core Domain Bound to a Full Consensus Site as a Self-Assembled Tetramer. *Structure* **18,** 246–256 (2010).
2. Joerger, A. C., Ang, H. C., Veprintsev, D. B., Blair, C. M. & Fersht, A. R. Structures of p53 cancer mutants and mechanism of rescue by second-site suppressor mutations. *J. Biol. Chem.* **280,** 16030–16037 (2005).
3. Berg, O. G., Winter, R. B. & Hippel, von, P. H. Diffusion-driven mechanisms of protein translocation on nucleic acids. 1. Models and theory. *Biochemistry* **20,** 6929–6948 (1981).

4. Anderson, M. E., Woelker, B., Reed, M., Wang, P. & Tegtmeyer, P. Reciprocal interference between the sequence-specific core and nonspecific C-terminal DNA binding domains of p53: implications for regulation. *Mol. Cell. Biol.* **17,** 6255–6264 (1997).
5. Mazza, D., Abernathy, A., Golob, N., Morisaki, T. & McNally, J. G. A benchmark for chromatin binding measurements in live cells. *Nucleic Acids Res.* **40,** e119–e119 (2012).

Figure legends:

Fig. S1

Calibration of a parameter for interaction between the Core and the RE. (A) The initial structure of the simulation. The system contains one Core (green) and one RE (purple) in a spherical boundary (transparent blue). (B) Typical time trajectories of nearest distance between the Core and the RE for three different parameter values ϵ in eq. S1. (C) Probability distributions of interaction energy between the Core and the RE. In (B) and (C), colors represents different ϵs (0.20 (red), 0.27 (green) and 0.29 (blue)). (D) Dissociation constants, K_ds, against ϵs.

Fig. S2

The probability distributions of nearest distance between p53 and dsDNA in the simulations for $p53_0$ (red) and $p53_n$ (blue).

Fig. S3

Two-dimensional probability distributions of the two angles, α and β, in the 1-Core bound state (A) and the 3-Core bound state. Refer to the text for the definitions of these angles.

Movie legends:

Supplementary Movie 1

A coarse-grained simulation trajectory of $p53_0$ on DNA strand with the recognition element. The color assignment is the same as that of Fig. 1. The simulation time in the movie is 300 μs.

Supplementary Movie 2

A coarse-grained simulation trajectory of $p53_n$ on DNA strand with the recognition element. The color assignment is the same as that of Fig. 1. The simulation time in this movie is 400 μs.

Supplementary Movie 3

A coarse-grained simulation trajectory of $p53_0$ on RE. The color assignment is the same as that of Fig. 1. The simulation time in this is 300 μs.

Figure S1

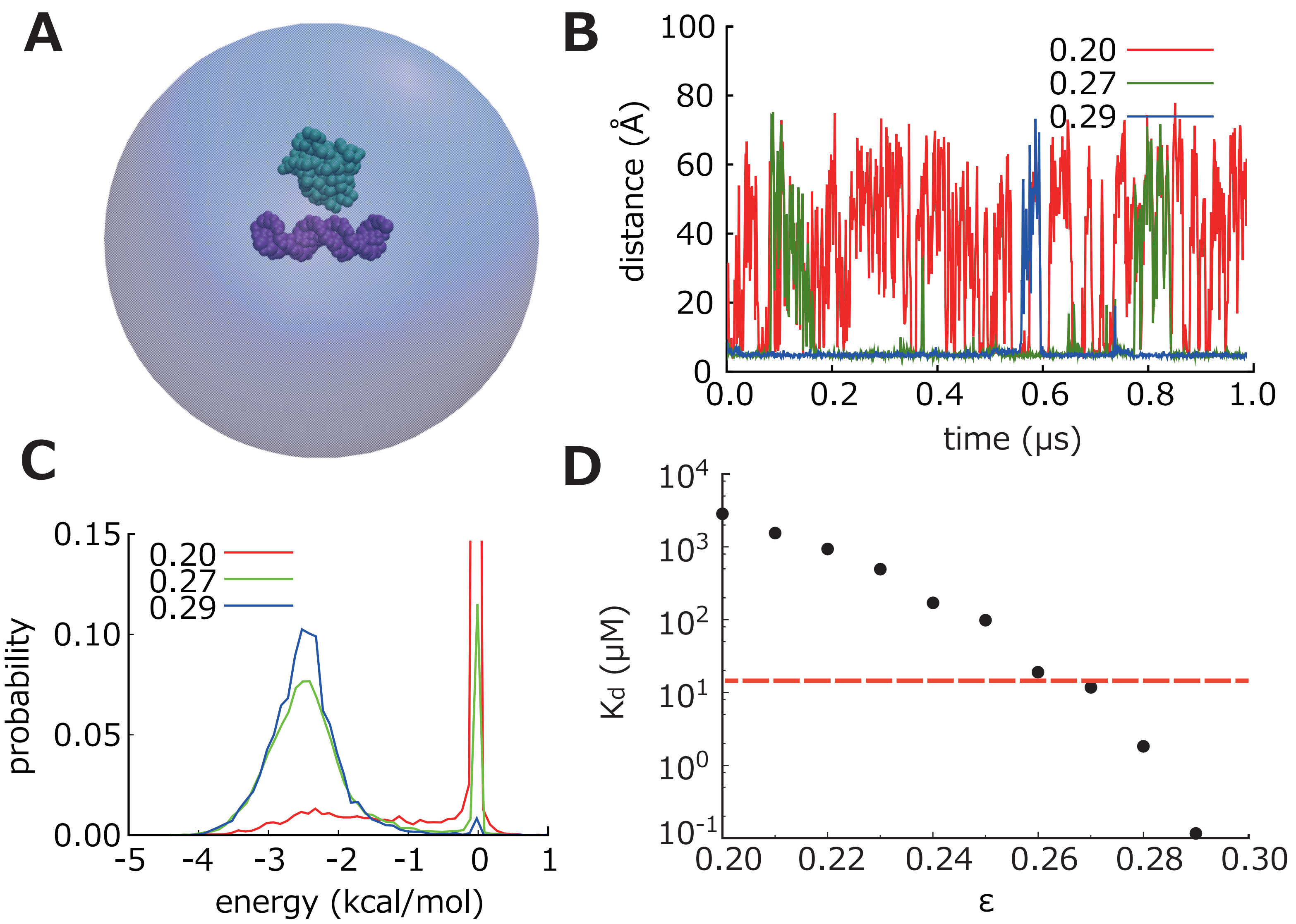

A
B
100
80
60
40
20
0
distance (Å)
0.0
0.2
0.4
0.6
0.8
1.0
time (μs)
0.20
0.27
0.29
C
0.15
0.10
0.05
0.00
probability
-5
-4
-3
-2
-1
0
1
energy (kcal/mol)
0.20
0.27
0.29
D
10^4
10^3
10^2
10^1
10^0
10^{-1}
K_d (μM)
0.20
0.22
0.24
0.26
0.28
0.30
ε

Figure S2

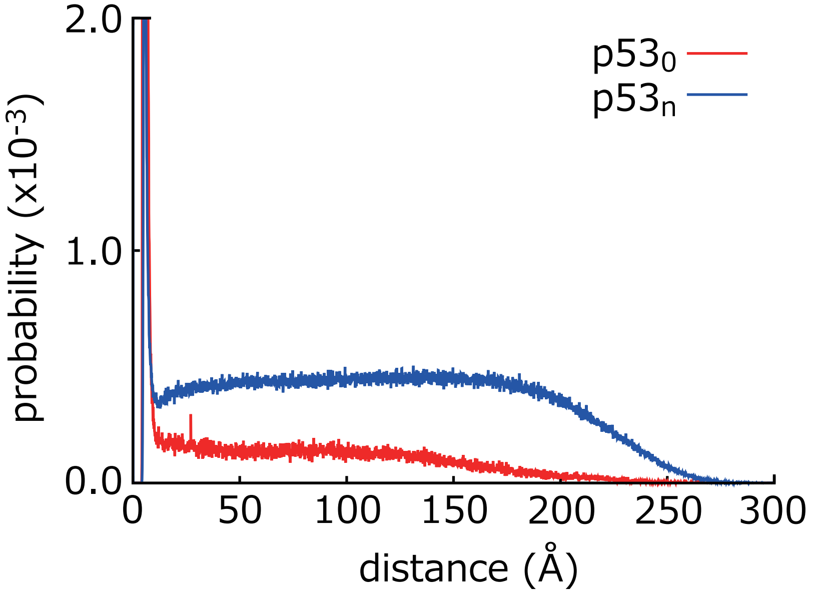

2.0
1.0
0.0
probability (x10-3)
0
50
100
150
200
250
300
distance (Å)
p53_0
p53_n

Figure S3

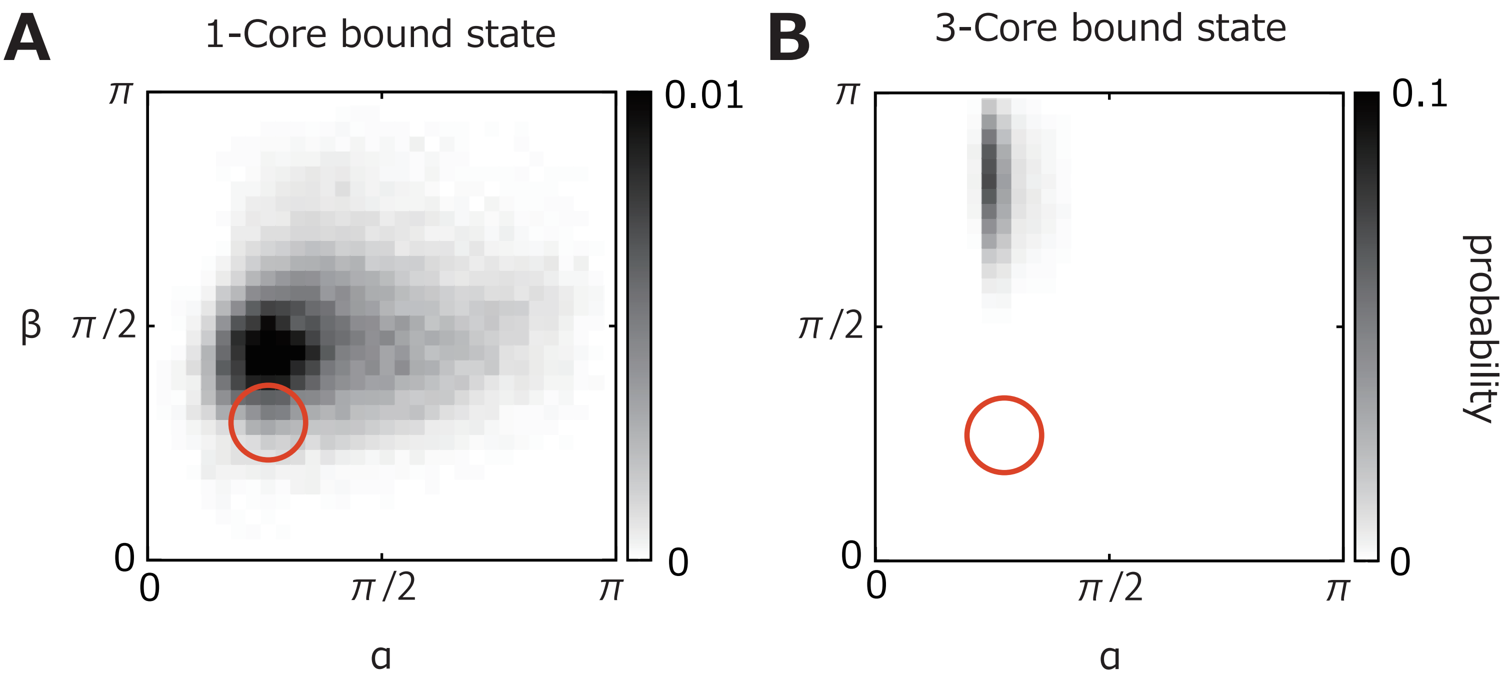

A
1-Core bound state
π
0.01
β π/2
0
0
0
π/2
π
α
B
3-Core bound state
π
0.1
π/2
probability
0
0
0
π/2
π
α
